# The Deviation of the Behaviors of Rice Farmers from Their Stated Willingness to Apply Biopesticides—A Study Carried Out in Jilin Province of China

**DOI:** 10.3390/ijerph18116026

**Published:** 2021-06-03

**Authors:** Hongpeng Guo, Fanhui Sun, Chulin Pan, Baiming Yang, Yin Li

**Affiliations:** 1 College of Biological and Agricultural Engineering, Jilin University, 5988 Renmin Street, Changchun 130022, China; ghp@jlu.edu.cn (H.G.); sunfh20@mails.jlu.edu.cn (F.S.); 2 Changchun Guoxin Modern Agricultural Science and Technology Development Co., Ltd., Shuangyang District, Changchun 130600, China; Ybm0431@163.com (B.Y.); 15243101004@139.com (Y.L.)

**Keywords:** biopesticides, rice, application willingness, application behaviors, behaviors deviate from willingness

## Abstract

The substitution of chemical pesticides by biopesticides is crucial to ensure the quality of agricultural products and to foster environmental sustainability. This study takes the willingness and the behaviors of rice farmers on the application of biopesticides as the research object. The survey questionnaire was designed based on the theory of rational small-scale farmers from three aspects: “individual and family characteristics of farmers”, “cognition of farmers” and “external factors”. The survey was then conducted on 163 rice farmers in seven prefecture-level cities in Jilin Province of China. The logistic model was used to analyze the influencing factors resulting in the deviation of the behaviors of the rice farmers from their initial willingness on the application of biopesticides. The explanatory structure model (ISM) was used to analyze the logical hierarchical relationship among various influencing factors. The results show that: (1) For 45% of the farmers surveyed, there’s a deviation between their willingness and behaviors regarding the application of biopesticides; (2) Among the significant factors leading to the deviation between farmers’ willingness and behaviors concerning the application of biopesticides, the surface-level direct factor is biopesticide awareness. The mid-level indirect factors are agricultural product quality and safety awareness and the deep-level root cause is farmers’ education level. (3) The primary reason for the deviation of the farmers’ behaviors from their willingness is their lack of knowledge about biopesticides and the biopesticides’ incomplete market structure. Based on the comprehensive analysis, it is recommended to improve the professionalization of the farmers, to strengthen the publicity of green production and to accelerate the formulation of the biopesticides market to further promote the usage of biopesticides.

## 1. Introduction

The amount of pesticides used globally to control crop pests and diseases is estimated to be around 6 million tons per year. The effective utilization rate of pesticides is less than 30% while the non-effective misusage is as high as 70% [1]. Residual pesticides in the environment spread rapidly under wind, rain and other meteorological conditions, leading to the condition where pesticide residues are trapped in the air, oceans, soil and organisms worldwide [1]. Since 2007, China has ranked no.1 in the world in terms of pesticide production and usage, however there’s also adequate evidence showing that overusage of pesticides is very common in China. According to statistics, the total amount of pesticides utilized in China in 2019 was still as high as 1,392,000 tons and the average dosage of pesticide application reached 8.39 kg/ha, which was higher than the internationally accepted upper limit of 7.5 kg/ha based on safety considerations [2]. The massive use of chemical pesticides will not place heavy pressure on the ecological environment, it will also adversely affect the quality and safety of agricultural products due to the potential presence of pesticide residues [3], Pesticide residues can also pose a serious threat to human being’s health through the food chain and the accumulation of bioconcentration effects [4]. Some member states of the European Union were the first to put forward and implement the concept of reducing pesticide usage to reduce the impact on the agroecological environment. Since then, the use of pesticides in several countries have shown a decreasing trend [5]. Similar policies on the reducing pesticide usage have also been implemented in Korea, Japan and other countries in Asia, where the usage of pesticides has decreased significantly in recent years [6]. However, the usage of pesticides in major countries in the Americas has still been growing rapidly, especially for herbicide usage [7]. To better tackle the problem of limited global agricultural resources and food safety issues, biopesticides have been more frequently used over traditional chemical pesticides because they control through natural substances or living organisms [8]. They also have the advantages of being flexible, less likelihood of producing resistance, harmless to plants, human beings, animals and the environment and they are eco-friendly products [9]. They are also the most important production inputs for organic agriculture and they a crucial role in agricultural sustainability [10]. In order to promote green agriculture and to increase agricultural product quality, it’s necessary to shift farmers’ traditional way of using chemical pesticides into using biopesticides [11]. Environmental protection and sustainable development are nothing new in China and majority of the Chinese farmers do have an expressed willingness to adopt green production techniques [12]. However, due to high production and preservation costs for biopesticides and the farmers’ lack of knowledge, the adoption level of biopesticides in China is still not promising. The market share of biopesticides usage stands at less than 10%, which is 50% lower than the world average [13]. Differences in the behaviors and willingness of farmers to apply biopesticides in actual agricultural productions have been observed [14]. Pray et al. found that more than one-third of agricultural producers in India expressed a willingness to use biopesticides, however only 3% of the farmers in the selected sample had actually used biopesticides in the past year [15]. Some scholars have noticed this phenomenon and it’s referred to as deviation or conflict between willingness and behaviors [16]. It will easily lead to wrong decisions from the government and enterprises on the production and promotion of biopesticides. Therefore, reducing the deviation of farmers’ behaviors from their willingness on the application of biopesticides is of vital importance to effectively promote the application of biopesticides and to realize the green transformation of agriculture.

Rice has been the predominant crop feeding 800 million people in China with a massive plantation area [17]. Rice has also played an important role in ensuring food security for the country. China is the largest producer of rice and, at the same time, China is also the largest consumer of rice. Jilin Province, as a major agricultural province in China, is an important rice production area in China [18]. Therefore, rice farmers in Jilin Province of China were chosen as the subjects of this paper. It is important to adopt biopesticides in the sustainable development of agriculture, especially in the current stage of low utilization rate of biopesticides. Through conducting research on rice farmers on their willingness and behaviors of biopesticides application and analyzing the influencing factors and the logical hierarchy among the influencing factors, the weak links in the promotion and the adoption of biopesticides in China at the present stage can be identified. It is also of great significance to alleviate the deviation of farmers’ behaviors from their initial willingness to apply biopesticides and to promote biopesticides more efficiently. It will also help to improve the competitiveness of the rice industry, to reduce environmental pollution and to gradually replace chemical pesticides with biopesticides. The study on the deviation of farmers’ willingness and behaviors to apply biopesticides in China will also be useful for the policy making of biopesticides in other developing countries.

### 1.1. Studies on Farmers’ Application Behaviors of Biopesticides 

Villa-Rodríguez et al. found that *Bacillus thuringiensis* (Bt), one of the major biopesticides used worldwide currently, was effective against rice leaf borer, stem borer, and stem borer [19]. In a survey conducted in U.S. farms, Wozniak concluded that the more educated one is, the more willing one is to try new things and take the risks they entail, and the more likely one is to embrace new technologies [20]. Paudel et al. concluded that the risk-averse psychology of growers affects the adoption of organic fertilizer technologies and that the degree of risk aversion (preference) of farmers affects their agricultural production and input behaviors [21]. Existing studies under the farmer perspective found that farmers’ personal characteristics, household characteristics [22], risk preference [23], technology perception [24], information ability [25], psychosocial perception [26] and production purpose [27] have significant impacts on farmers’ biopesticides purchase behaviors, their willingness to apply and their application behaviors. The government perspective focuses on the promotion role of policy guidance [28] as well as government publicity and education [29] in the adoption process of biopesticides by farmers.

### 1.2. Studies on the Deviation of Behavioral Intentions 

Most of them are based on the Theory of Rational Behavior (TRA) or the Theory of Planned Behavior (TPB) [24,27,30], using methods such as structural equations to analyze the influence of behavioral attitudes [31], perceptual behavioral control [32], subjective norms [33] and other dimensions on the divergence of some behavioral intentions and applied behaviors [31]. The research area is mainly focused on the product consumption of individuals. Relevant studies in the field of agriculture are fewer, with research topics scattered widely. Theoretically, Icek argued that willingness is a condition of the process of achieving the desired behavioral goal and it’s predictive [34]. Meanwhile Newman argued that willingness and behavior can show inconsistencies, either in the form of a blocked conversion of willingness into behaviors or in the form of a deviation of behaviors from the initial willingness due to external interference, and that willingness will not effectively convert behaviors [35]. Waithaka argued that deviation is influenced by internal endogenous drivers and external situational changes [36]. Jeffrey R. found that the theory of planned behavior adds to the individual’s subjective willingness the conditions and ability to perform a specific behavior [37], and since the ability to perform and subjective willingness are collectively referred to as perceived behavioral control, perceived behavioral control can directly influence individuals’ behavioral intentions and applied behaviors. To date, most of the relevant studies on the behavioral analysis of farm household are based on the willingness-behavior deviation perspective focusing on new rural cooperative medical care [38], food security [39] and small-scale water constructions [40].

The existing international research findings on biopesticides show that the relevant literature started earlier and the topic has been studied deeply. However, there are few studies on the behaviors of farmers on the application of biopesticides, especially for rice farmer households in Northern China. There is a gap in the research on the deviation of behaviors of rice farmers from their willingness to apply biopesticides and the mechanisms and factors influencing the deviation of biopesticide application decisions need to be analyzed.

Therefore, this study uses a logistic regression model to empirically analyze the factors influencing the divergence between the willingness and behaviors of the rice farmers on the application of biopesticides. Theoretical support and practical guidance are pro-vided for better and prompt promotion of biopesticides and improved utilization rate of biopesticides.

## 2. Materials and Methods

### 2.1. Data Source

The data used in this study were obtained from survey questionnaires and interviews conducted by the research team from October to December 2020 among rice farmers in Jilin Province of China. A multi-stage random sampling method was used to select the samples during the actual survey [41]. First, based on the scale of rice cultivation, a total of seven counties and cities were selected in Jilin Province, including Changchun, Jilin, Siping, Liaoyuan, Tonghua, Songyuan, and Baicheng. Then, two townships were randomly selected in each county and city. Finally, three natural villages were randomly selected in each township, and 3–8 rice growing households were randomly selected in each village for our questionnaire survey. Data were obtained on individual characteristics, household characteristics, knowledge of biopesticides, willingness to apply biopesticides in rice production and their application behaviors, together with other related variables. We issued a total of 200 questionnaires, and the focus of this study is on the difference between the willingness and behavior of biopesticide application, i.e., farmers who have the willingness but do not have the behavior, so the questionnaires of farmers without the willingness to apply biopesticides were excluded. After the later research error checking and sorting, questionnaires were excluded that are invalid or farmers who don’t have the willingness, and finally 163 valid and willing farmers’ questionnaires were obtained, with an effective rate of 81.5%. The basic information of the farmers surveyed is shown in Table 1.

Descriptive statistics of the samples show that: the age of the farmers surveyed was mainly between 41 and 50 years old (43.6%), with an average age of 45.88 years old; 73.6% of them were men and 26.4% were women; 42.9% of the farmers participated in cooperatives; the education level of farmers were mainly primary and junior high school, accounting for 74.8% of the total number of samples; the average scale of rice cultivation was 18.3 mu; the average annual household income was 118,600 yuan; the proportion of income from rice plantation mainly ranged from 80% to 100% (33.1% of them) with an average value of 78.75%. 

Therefore, most of the farmers being surveyed were middle-aged males with a higher proportion of household income from planting rice. It’s also worth mentioning that a majority of them have an education level of junior high school and below. Jilin Province is an important rice production area in China and this paper uses stratified random sampling from dispersed geographical locations for the survey to ensure the samples selected are representative in serving the research needs for this study.

### 2.2. Variable Settings

Based on the theoretical basis of the rational smallholder theory proposed by Schultz [42] in combination with related studies [43], we analyzed the factors leading to the divergence between farmers’ willingness to apply biopesticides and their behaviors from three aspects: farmers’ individual and family characteristics, farmers’ perceptions and external factors. Based on the construction of the theoretical model, “farmers’ willingness and behaviors to apply biopesticides” was set as the dependent variable and “factors influencing the deviation of farmers’ behaviors from willingness to apply biopesticides” as the independent variable, including farmers’ individual and family characteristics, farmers’ perceptions and external factors. 

#### 2.2.1. Dependent Variable

Referring to the existing scholars’ measures [30,31,37], this paper defines the deviation between the farmers’ willingness and behaviors to apply biopesticides as a phenomenon in which farmers show willingness to apply biopesticides in the agricultural production process without taking actual actions. In other words, there’s inconsistency shown between their willingness and behaviors. Based on this definition, the samples of this paper should be those farmers who have the initial willingness to apply biopesticides in their agricultural production process. Statistical analysis shows that 163 farmers out of 200 samples have the willingness to apply biopesticides, hence this paper will conduct empirical analysis based on these 163 samples. For those farmers that don’t have biopesticide application behaviors, deviation exists and y = 1; if farmers have biopesticide application behaviors, there’s no deviation and hence y = 0. The details are shown in Table 2.

#### 2.2.2. Independent Variable

(1) Individual and Family Characteristics

Numerous studies have shown that both individual characteristics of farmers and their family characteristics pose an impact on their deviation between behaviors and willingness. The individual characteristics of farm households mainly refer to gender, age, and education level of the farmers surveyed. Gender differences are reflected in the decision-making process in agricultural production. Different scholars have different opinions on this issue. Some of the scholars’ studies concluded that male farmers usually have higher exposure to the outside world than women with a better understanding of pesticides and they have a better awareness of the associated health risks from using chemical pesticides [3,44]. However, some other scholars believed that women are more concerned about their own safety and health than men in the process of pesticide application [45,46]. Binswanger et al. showed that younger farmers are more inclined to take risks [47], while older farmers, who may have developed an empirical dependence on chemical pesticides during their long-term agricultural practices, are more inclined to choose chemical pesticides [48]. The education level of farmers reflects, to some extent, their ability to obtain in-formation and to acquire skills. Farmers with higher education level are more likely to adopt biopesticides [49].

The agricultural households’ business characteristics of farmers mainly include whether they participate in cooperatives, annual household income, percentage of annual income from rice plantation and scale of rice cultivation. Farmer cooperatives are an important part of the agricultural science and technology extension system and they play an crucial role in the promotion of biopesticides [50]. Therefore, farmers who participate in farmers’ cooperatives are generally aware of modern agricultural productions and thus they know the advantages of environmentally friendly agricultural technologies. So, it is more likely that they will choose biopesticides over conventional chemical pesticides [51]. Biopesticides are more expensive compared with chemical pesticides. The higher the income level of farmers’ households, the higher their probability of putting in more investment and adopting biopesticides [52]. The percentage of the farmers’ household’s annual income from rice plantation measures the dependence of farmers on land. By considering opportunity cost, the lower the percentage of their annual income out of rice plantation, the less likely they are to choose biopesticides [53]. Related studies have shown that planting scales have a facilitating effect on rice farmers’ biopesticide application behaviors [54] and large-scale households tend to have more social capital and human capital compared with smaller farmer households hence they have better access to external resources [55]. This implies that farmers with a large cultivation scale have more human and financial resources, pest control expertise as well as broader information access, so they are inclined to choose biopesticides for early prevention and disease control at the right timing [56].

Therefore, this paper hypothesized that farmers’ education level, annual household income and rice cultivation scales have a negative effect on the deviation of farmers’ willingness and behaviors to apply biopesticides. While farmers’ age, participation in cooperatives and percentage of income out of rice plantation have a positive effect on the deviation of farmers’ willingness and behaviors to apply biopesticides. The gender of the farmer has an uncertain influence on the deviation of farmers’ willingness and behaviors to apply biopesticides.

(2) Farmers’ Awareness

Farmers’ perceptions are one of the most important factors affecting farmers’ choices of pesticides [48]. Farmers’ perceptions of pesticides mainly include their understanding of the characteristics of biopesticides, the hazards posed on human health and environmental pollution from long-term application of chemical pesticides, their concern about the quality of agricultural products and their confidence level in the efficiency of biopesticides. Farmers are more inclined to choose biopesticides over chemical pesticides if they recognize that long-term application of chemical pesticides will do harm to human beings’ health and bring about environmental problems such as soil acidification, soil caking and nutrient decline [29]. The better the farmers’ awareness of effectiveness of biopesticides and the more serious they are with the quality and safety of agricultural products, the greater the possibility that they will choose biopesticides over chemical pesticides [57]. In contrast, if farmers do not believe in the promotional effects of biopesticides and the more skeptical they are about biopesticides, the more likely they will be driven to abandon the adoption of biopesticides [58].

Therefore, this paper hypothesized that the farmers’ knowledge about the characteristics of biopesticides, their awareness about the environmental pollution caused by the long-term application of chemical pesticides and their concern over the quality and safety of agricultural products have a negative influence on the deviation of farmers willingness and behaviors to apply biopesticides. On the contrary, farmers’ skepticism about the effectiveness of biopesticides has a positive influence on the deviation of their behaviors from willingness on the application of biopesticides.

(3) External Factors

Different external factors have different impacts on the deviation of willingness and behaviors. Farmers tend to abandon the application of biopesticides themselves when there’s no one around them applying biopesticides [26]. The prerequisite for farmers to apply biopesticides is the availability of adequate biopesticides [59]. In the case of unexpected outbreak of severe pests’ diseases in the fields, farmers will be more likely to choose chemical pesticides that are fast-acting and easily accessible [60]. When purchasing pesticides, farmers will consider the price of the pesticides. Commercial biopesticides are generally more expensive, which may lead to farmers’ reluctance to apply biopesticides [61]. Therefore, this paper hypothesized that peer influences, emergency conditions, market availability and price affordability all have positive effects on the deviation of farmers’ willingness and behaviors to apply biopesticides from the farmers’ perspective.

Therefore, the independent variable including 15 factors in 3 areas. The specific variable definitions and their descriptive statistics are shown in Table 3.

### 2.3. Research Methodology

A deeper analysis of the logical hierarchy among the influencing factors is of great theoretical and practical significance in studying the correlation between farmers’ willingness and behaviors in biopesticide application. Therefore, in this paper, a logistic regression model has been chosen to filter the influencing factors. Moreover, the hierarchical relationships among influencing factors have been analyzed using the ISM model [30,31,32,37,62].

#### 2.3.1. Logistic Regression Model

This study investigates the factors affecting the deviation of rice farmers’ behaviors from their initial intentions of biopesticide application. The dependent variable is “whether biopesticide application intentions and behaviors deviate from each other”, which is a typical binary decision problem, i.e., “deviation” and “non-deviation”. Therefore, in this paper, a logistic regression model has been chosen to investigate the factors influencing the deviation of farmers’ behaviors from their initial willingness in biopesticide application [63,64,65]. For those farmers that don’t have biopesticide application behaviors, deviation exists and y = 1; if farmers have biopesticide application behaviors, there’s no deviation and hence y = 0. The logistic regression model is as follows:

(1) $$P_{i}=F\left(y_{i}\right)=\left(\beta_{0}+\overset{n}{\underset{j=1}{\sum }}\beta_{j}X_{ij}\right)=\frac{\exp\left(\beta_{0}+\sum_{j=1}^{n}\beta_{j}X_{ij}\right)}{1+\exp\left(\beta_{0}+\sum_{j=1}^{n}\beta_{j}X_{ij}\right)}$$

where $P_{i}$ is the probability of deviation between the application intentions and behaviors of farmer *i*; $F\left(y_{i}\right)$ is the probability distribution function; $\beta_{0}$ is the intercept term; $\beta_{j}$ is the regression coefficient of the *j*-th independent variable; *n* is the number of independent variables; $X_{ij}$ is the value of the *j*-th variable of the *i*-th farmer.

By taking the logarithm of both sides of Equation (1), the simplified form is obtained as:

(2) $$y_{i}=ln\left(\frac{P_{i}}{1-P_{i}}\right)=\beta_{0}+\overset{n}{\underset{j=1}{\sum }}\beta_{j}X_{ij}$$

#### 2.3.2. ISM Model

The factors influencing the divergence between farmers’ willingness to apply biopesticides and their behavior in rice cultivation are both independent and interrelated, and it is important to distinguish the hierarchy of relationships among the factors to identify the key reasons for the divergence between willingness and behavior, and even to solve the problem of biopesticide promotion efficiency [37]. Therefore, this paper further analyzes the correlation and hierarchy between the factors influencing the divergence between farmers’ willingness to apply biopesticides and their behavior by using the ISM model [62,66]. The steps of the ISM model are as follows [67]:

##### Determine the Adjacency Matrix R between the Factors

Assuming that there are k significant influencing factors; $S_{0}$ is the deviation of farmers’ intentions to apply biopesticides from their behaviors; $S_{i}\left(S_{j}\right)$ denotes the $i\left(j\right)$ significant influencing factor; the components of the adjacency matrix R are defined by Equation (3):

(3) $$r_{ij}=\left\{\begin{array}{c} 1\;\left(S_{i}\;\mathrm{is}\;\mathrm{related}\;\mathrm{to}\;S_{j}\right)\\ 0\;\left(S_{i}\;\mathrm{is}\;\mathrm{not}\;\mathrm{related}\;\mathrm{to}\;S_{j}\right) \end{array}\right.\;\left(i=0,1,\ldots ,k;\;j=0,1,\ldots ,k\right)$$

Determine the reachable matrix M among the factors, which is calculated from Equation (4)

(4) $$M={\left(R+I\right)}^{\lambda +1}={\left(R+I\right)}^{\lambda }\neq {\left(R+I\right)}^{\lambda -1}\neq \ldots \neq {\left(R+I\right)}^{2}\neq \left(R+I\right)$$

where *I* is the unit matrix, 2≤λ≤k and the Boolean operator is used in the power operation of the matrix.

##### Determine the Hierarchy of Each Factor

According to Equation (5), the reachable matrix is divided into the reachable set $P\left(S_{i}\right)$ and the antecedent set $Q\left(S_{i}\right)$ and both represent the set of all factors in the reachable matrix that can be reached from the factor $S_{i}$, where both $m_{ij}$ and $m_{ji}$ represent the factors in the reachable matrix. Equation (6) determines the highest level $\left(L_{i}\right)$ and its influencing factors, as well as the other levels of factors. To do this, we remove the rows and columns of the highest-level factors from the reachable matrix M to form the reachable matrix. By repeating the steps in Equations (5) and (6), the factors at the second level and all other levels can be obtained:

(5) $$\;P\left(S_{i}\right)=\left\{S_{j}\left|m_{ij}=1\right.\right\},Q\left(S_{j}\right)=\left\{S_{j}\left|m_{ji}=1\right.\right\}$$

(6) $$L_{i}=\left\{\left.S_{i}\right|P\left(S_{i}\right)\cap Q\left(S_{i}\right)=P\left(S_{i}\right);i=0,1,\ldots ,k\right\}$$

Determine the Hierarchical Structure of Each Influencing Factor.

Directional arrows have been used to connect factors between adjacent levels and at the same level to obtain a hierarchical structure of all the influencing factors.

## 3. Results

### 3.1. Logistic Regression Results

Before adopting the logistic regression model, the possible multicollinearity in the explanatory variables was firstly diagnosed by the multicollinearity test and the results showed that the variance inflation factors (VIF) were all less than 10, indicating that there’s no multicollinearity among the variables. Based on that, the regression analysis of the sample data was performed using Stata software and the results are shown in Table 4.

### 3.2. ISM Analysis Results

**Figure 1:**
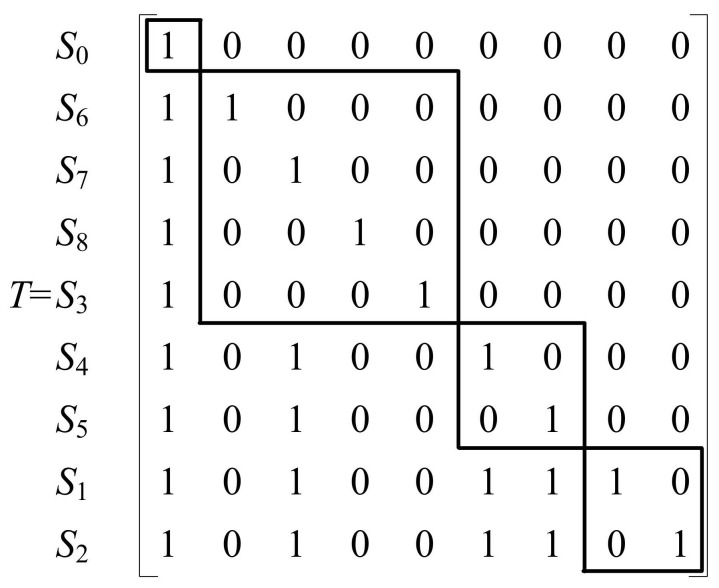
Driving Factor Hierarchy T Diagram.

As seen from the regression results of the logistic model in Table 4, eight significant factors are influencing the deviation of behaviors and willingness of biopesticide application of rice farmers and the systematic composition of the deviation is determined as S_i_ = (S_1_, S_2_, …, S_8_), representing education level, scales of planting, biopesticide awareness, awareness of hazardous effect from chemical pesticides, quality and safety awareness of agricultural products, peer influences, emergency conditions and price affordability, respectively. The willingness to apply biopesticides and behavioral deviations are represented by S_0_. According to the ISM explanatory structure model, the Matlab matrix operation tool was applied to obtain the hierarchical structure T of the factors influencing the willingness to apply biopesticides and behavioral deviations of rice farmers. The box indicates the same level of factors as shown in Figure 1.

## 4. Discussion

### 4.1. Analysis of the Factors Influencing the Deviation of Biopesticide Application Intentions and Behaviors of Rice-Growing Farmers

#### 4.1.1. Analysis of the Impacts of Individual and Family Characteristics

The comprehensive regression results show that both education level and scales of rice planting pass the 5% significance test hence both have a significant negative effect on the deviation of willingness and behaviors of biopesticide application. It’s confident to conclude that the higher the education level of the farmers and the larger the scales of rice planting, the less likely there’s deviation between their behaviors and willingness. This is because the farmers with better education level have a deeper understanding of biopesticides, so it’s the easier for them to adopt the application of biopesticides. At the same time, for those farmers having larger scales of rice plantation, agricultural production has become their major work. Since the application of biopesticides can effectively ensure the smooth implementation of agricultural production, their behaviors and willingness to apply biopesticides are less likely to diverge.

#### 4.1.2. Analysis of the Influences from Farmers’ Perceptions

Biopesticide awareness has a significant negative effect on the deviation of farmers’ willingness and behaviors of biopesticide application and it’s at 10% significance level. Combined with the results from descriptive statistics, there’s lack of knowledge of biopesticides currently for most of the farmers. By taking into consideration their age and low education level, they hardly search for information about biopesticides actively. Although farmers claim that they are willing to apply biopesticides, they have difficulties in appreciating the advantages of biopesticides due to their lack of knowledge and expertise about them and therefore they tend to give up transforming their willingness into concrete behaviors.

The awareness of hazardous effects of chemical pesticides and quality and safety awareness of agricultural products are negatively correlated with the deviation of willingness and behaviors of biopesticide application, with both of the factors are statistically significant at 5% level. The reason is that, on one hand, farmers with a higher level of awareness of environmental pollution and the hazards of chemical pesticides are usually better educated and relatively younger. The more farmers willing to pay attention to the protection of rural ecological environment and health, the higher the chance they will adopt the usage of biopesticides. So, the likelihood of deviation of their behaviors from willingness on the application of biopesticides is lower. On the other hand, quality and safety awareness of agricultural products is part of social responsibility and it’s also the farmers’ own psychological initiative to protect rural ecological environment. Therefore, the stronger the awareness towards agricultural products’ quality and safety, the less likely the divergence between farmers’ willingness and behaviors on biopesticides application will take place.

#### 4.1.3. Analysis of the Influences from External Factors

Peer influences have a significant positive effect on the deviation of farmers’ willingness and behaviors in biopesticide application and it’s at 1% significance level. It means that farmers tend to rely heavily on neighboring farmers when it comes to the procurement and application of pesticides. Peer influences can also be referred to as social customs or social norms. Farmers are pressured by social norms to conform to the behavioral expectations of others when conducting agricultural productions. In the survey, it was found that many farmers were willing to apply biopesticides at the first place, however the intention was abandoned as the neighboring farmers were still using chemical pesticides.

The emergency conditions have a significant positive effect on the divergence between farmers’ willingness and behaviors to apply biopesticides and it has passed the 5% significance test. Farmers often face the dilemma of whether purchasing and applying the highly toxic but fast-reacting chemical pesticides or sticking with environmentally friendly biopesticides. Emergency conditions refer to the temporary anxiety, excitement, and tensions that farmers show in their daily agricultural productions [68]. Although a certain degree of contingency exists, it can still affect farmers’ environmental perceptions by changing their own emotions, which in turn affects the deviation of farmers’ willingness and behaviors in biopesticide application.

The price affordability has a significant positive effect on the divergence between farmers’ willingness and behaviors of biopesticide application and it has passed the 1% significance test. It indicates that the price of pesticides is still one of the most crucial factors that farmers consider when purchasing. This is because farmers are still mainly rational people and profit optimization is the goal of conducting agricultural productions. Although most farmers are aware of environment protection and they have showed their willingness to apply biopesticides, the higher price of biopesticides discourages farmers in the end. When farmers’ willingness to protect the environment conflicts with the high purchasing price, majority of them will opt for cheaper options, which is chemical pesticides.

### 4.2. Hierarchical Analysis of the Factors Influencing the Deviation of Biopesticide Application Intentions and Behaviors of Rice-Growing Farmers

**Figure 2:**
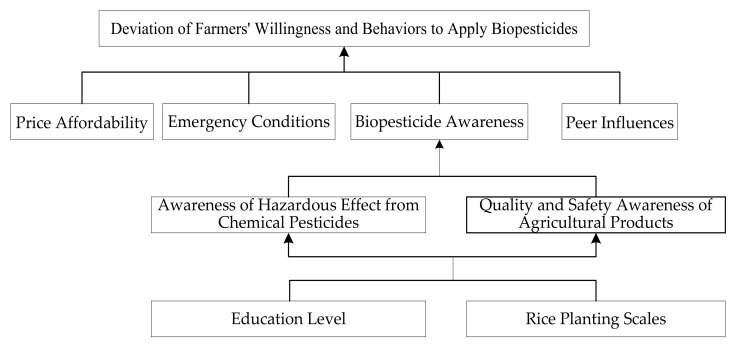
Interpretative Structural Model of Influencing Factors.

Based on the results of logical hierarchical analysis (Figure 2), it is observed that the influencing factors are in different hierarchical structures, which are both independent from and interrelated with each other. Price affordability, emergency conditions, peer influences and biopesticide awareness are the direct influencing factors at the surface level. Awareness of hazardous effects from chemical pesticides and quality and safety awareness of agricultural products are the indirect factors at the mid-level while education level and cultivation scales are the root causes. The logical hierarchy among these factors can be summarized as a “single path with three drivers” model. The reason for this hierarchy to appear is mainly because that the application of biopesticides is determined by farmers but at the same time it’s constrained by realistic situations.

Single path: education level, scales of rice planting → awareness of hazardous effects from chemical pesticides, quality and safety awareness of agricultural products → biopesticide awareness → farmers’ willingness to apply biopesticides and behavioral deviation.

In this pathway, farmers’ individual and business characteristics such as farmers’ education level and planting scales are the most fundamental driving forces. Their awareness of hazardous effects from chemical pesticides and their awareness of quality and safety of agricultural products are the external manifestations of the root factors. Intermediate factors will further influence farmers’ perception of biopesticides, which in turn directly affects the deviation of farmers’ willingness and behaviors to apply biopesticides. Farmers’ actual behaviors are influenced by farmers’ perceptions, which are derived from their own perceptions of the hazards that chemical pesticides pose and their sense of responsibility to protect the quality and safety of agricultural products. Such perceptions and awareness of the environment protection reflect the personal characteristics of farmers and the operational characteristics of agricultural productions as well.

Three drivers: price affordability, emergency conditions, peer influences → deviation of farmers’ willingness and behaviors to apply biopesticides.

Farmers, as the most important group of people in agricultural productions, will face a variety of realistic scenarios when choosing pesticides. They need to consider the effectiveness of pesticides and the reaction time; they will also conduct a comparative analysis of various inputs and outputs to choose the most cost-effective production methods. At the same time, they are also heavily relying on their neighboring farmers as for the selection and application of pesticides.

## 5. Conclusions

The analysis in this study was based on data from sample surveys of rice-growing farmers in seven prefectural-level cities in Jilin Province of China from October to December 2020. Logistic-ISM model has been used to analyze the key factors influencing the deviation of rice growing farmers’ willingness and behaviors to apply biopesticides and the logical hierarchy among the key factors have been analyzed in depth:

(1) There are still many farmers using chemical pesticides and there are many deviations between their willingness and behaviors in the application of biopesticides, so it is not promising to promote biopesticides as an alternative to chemical pesticides on a full scale. The divergence between the willingness and behaviors of rice farmers to apply biopesticides is influenced by various factors. In terms of individual and family characteristics, both education level and scales of rice planting have a negative effect on it. In terms of farmers’ awareness, biopesticide awareness, awareness of hazardous effects from chemical pesticides and quality and safety awareness of agricultural products have a negative effect on the deviation. The better the farmers’ awareness towards environment protection, the lower the possibility of the deviation to take place. As for external factors, peer influences, emergency conditions and price affordability have positive effects on the occurrence of deviation.
(2) The logical hierarchy of influencing factors can be summarized as a “single path with three drivers” model. Biopesticide awareness is at the surface level, awareness of chemical pesticides’ hazards and awareness of agricultural quality and safety are indirect factors at the mid-level while the farmers’ characteristics such as education level and planting scales are root causes. The three drivers refer to external factors such as price affordability, emergency conditions and peer influences and they are also surface-level direct influencing factors. It’s very important for governing agencies to put focus on these root causes while promoting the application of biopesticides to achieve a promising outcome.
(3) Some of the key reasons for the deviation of farmers’ willingness and behaviors are listed below: farmers’ education level is generally low, farmers are not much concerned about the quality and safety of agricultural products, farmers’ lack knowledge and expertise about the characteristics of biopesticides and the hazardous effects from chemical pesticides. Farmers are constrained by economic conditions and their purchasing power is quite limited in terms of biopesticides procurement. In addition, the lack of publicity and incomplete construction of markets for biopesticides have led to farmers having difficulties distinguishing between biopesticides and chemical pesticides.

### Suggestions

Through the formulation of policy and measures on major influencing factors, the conversion of willingness to behaviors can be improved hence reducing the deviation of behaviors from willingness. A few suggestions have been made to relevant departments and local governments based on the results from this study:

(1) It is extremely important to improve the expertise level of the farmers, to reduce the constraints of farmers’ resource endowment and to promote the conversion of farmers’ willingness to apply biopesticides into behaviors. It’s also necessary to enhance the education level of rural farmers through face-to-face coaching sessions and education on fields for farmers with low education level. In this approach, a new generation of young professional farmers can be cultivated with better agricultural expertise level. The promotion of biopesticides should also be focused such as the development of differentiated promotion programs for farmers of different planting scales in different regions.
(2) It is also recommended to strengthen the publicity of the ideas of green production and to raise the cognition level of farmers towards green production. On one hand, publicity and promotion work for biopesticide popularization through television, Internet and other social medias and face-to-face coaching can strengthen farmers’ understanding of green production and green transformation of agricultural production. On the other hand, it is necessary to deepen the farmers’ perceptions of green agricultural production experiences by carrying out special environmental protection activities such as organizing visits to green production demonstration projects and establishing green production demonstration households. In this way, farmers’ sense of responsibility to protect the environment in agricultural productions can be improved.
(3) Finally, it is crucial to speed up the establishment of the biopesticide market and to optimize the policy mechanisms and enforcement of biopesticide use. At present, farmers are facing the problem of selecting from various types of pesticides, which makes it difficult for farmers to distinguish between biopesticides and chemical pesticides. This phenomenon reminds us that attention should be paid to improving the identifiability of biopesticides at pesticide distribution sites hence reducing the extra identification costs for farmers. At the same time, price affordability is also one of the major concerns of farmers. The price of biopesticides need to be regulated to a relatively acceptable range through improved subsidy schemes and promotions. Moreover, subsidy schemes and promotions need to be made known to the public to obtain satisfaction from the farmers in order for them to have confidence in the application of biopesticides.

## Figures and Tables

**Table 1:** Basic Characteristics of the Samples.

Type	Options	Sample Size	Percentage (%)
Gender	Male	120	73.6
	Female	43	26.4
Age	≤30 years old	6	3.7
	31–40 years old	36	22.1
	41–50 years old	71	43.6
	51–60 years old	46	28.2
	>60 years old	4	2.5
Participation in Cooperatives	Yes	70	42.9
	No	93	57.1
Education Level	Below Primary School	2	1.2
	Primary School	38	23.3
	Junior High School	84	51.5
	High school or Junior College	27	16.6
	College and Above	12	7.4
Rice Revenue Share	0–20%	44	27.0
	20–40%	30	18.4
	40–60%	20	12.2
	60–80%	15	9.2
	80–100%	54	33.1

**Table 2:** Pesticide Application by Farmers.

Pesticide Application	Number of Samples (pcs)	Percentage (%)
Willingness Without Behaviors	73	45
Willingness with Behaviors	90	55
Total	163	100

**Table 3:** Variables of the Model and Descriptive Statistics.

		Variables	Variable Interpretation and Assignment	Average Value	Standard Deviation	Index Sources
Dependent Variable		Biopesticide Application Intentions and Behaviors	Deviation exists between intentions and actions. Yes = 1; No = 0	0.45	0.499	[37]
Independent Variables	Individual and Family Characteristics	Gender	Male = 1; Female = 0	0.74	0.442	[44]
		Age	30 years old and below = 1; 31–40 years old = 2; 41–50 years old = 3; 51–60 years old = 4; 60 years old and above = 5	3.04	0.867	[48]
		Education Level	Below elementary school = 1; Elementary school = 2; Junior high school = 3; High school or junior college = 4; College and above = 5	3.06	0.862	[49]
		Participation in Cooperatives	Do you participate in a cooperative? Yes = 1; No = 0	0.43	0.497	[51]
		Annual Household Income	Real annual household income/Ұ in 2019	11.86	0.500	[52]
		Percentage of Income from Rice Plantation	Rice revenue to total revenue ratio (%)	78.75	0.500	[53]
		Rice Planting Scales	Rice growing area (hm^2^)	1.22	0.500	[54]
	Farmers’ Awareness	Biopesticide Awareness	Do you know anything about biopesticides? Not at all = 1; Not very well informed = 2; General knowledge = 3; Well informed = 4; Very well informed = 5	2.54	0.897	[57]
		Awareness of Hazardous Effect from Chemical Pesticides	Are you aware of the hazards of chemical pesticides to humans and to the environment? Not at all = 1; Not very well informed = 2; General awareness = 3; Well informed = 4; Very well informed = 5	3.067	1.0548	[29]
		Quality and Safety Awareness of Agricultural Products	Are you concerned about the quality and safety of agricultural products? Not at all = 1; Not too concerned = 2; Generally concerned = 3; Much concerned = 4; Very much concerned = 5	3.71	1.094	[57]
		Confidence Level over Biopesticides Promotion	Do you believe in the effectiveness of biopesticides as advertised? Strongly disbelieve = 1; Relatively disbelieve = 2; General confidence level = 3; Relatively believe = 4; Strongly believe = 5	3.09	1.029	[58]
	External Factors	Peer Influences	The types of pesticides you would purchase are easily influenced by the farmers around you. Strongly disagree = 1; Relatively disagree = 2; General attitude = 3; Relatively agree = 4; Strongly agree = 5	3.75	0.810	[26]
		Emergency Conditions	When there’s outbreak of pest’s diseases, you would give priority to chemical pesticides. Strongly disagree = 1; Relatively disagree = 2; No preference = 3; Relatively agree = 4; Strongly agree = 5	3.82	0.925	[60]
		Biopesticides Availability	When you want to buy biopesticides, you cannot get it in time. Strongly disagree = 1; Relatively disagree = 2; No preference = 3; Relatively agree = 4; Strongly agree = 5	3.40	0.843	[59]
		Price Affordability	You think biopesticides are too expensive. Strongly disagree = 1; Relatively disagree = 2; Fair = 3; Relatively agree = 4; Strongly agree = 5	3.25	0.928	[61]

**Table 4:** Simulation Results of Regression Model.

Variable Category	Variable Name	Regression Coefficient *β*	Inspection Error S. E.	Power Value Exp (*β_i_*)
Individual and Family Characteristics	Gender	0.377	0.181	1.457
	Age	0.008	0.011	1.065
	Education level	−0.547 **	0.240	0.579
	Participation in Cooperatives	−0.248	0.328	0.781
	Annual Household Income	−0.015	0.163	0.985
	Percentage of Income out of Rice Plantation	0.112	0.162	1.189
	Scales of Rice Planting	−0.050 **	0.046	0.951
Farmers’ Awareness	Biopesticide Awareness	−0.507 *	0.221	0.602
	Awareness of Hazardous Effect from Chemical Pesticides	−0.710 **	0.173	0.492
	Quality and Safety Awareness of Agricultural Products	−0.936 **	0.315	0.392
	Confidence Level over Biopesticides Promotion	−0.192	0.210	0.825
External Factors	Peer Influences	1.702 ***	0.605	5.484
	Emergency Conditions	0.733 **	0.343	2.081
	Biopesticides Availability	0.284	0.425	1.287
	Price Affordability	0.385 ***	0.343	1.470
−2 times the log likelihood value		285.592		
R2 test		84.773 ***		

Note: ***, ** and * indicate that the coefficients of the explanatory variables are significant at the 1%, 5%, and 10% levels, respectively.

## Data Availability

Data sharing not applicable.
